# Acute disseminated encephalomyelitis following the COVID-19 vaccine Ad26.COV2.S, a case report

**DOI:** 10.1186/s42269-023-00981-7

**Published:** 2023-01-09

**Authors:** Stefan Gustavsen, Mette Maria Nordling, Arkadiusz Weglewski

**Affiliations:** 1 Department of Neurology, Copenhagen University Hospital-Herlev and Gentofte, Borgmester Ib Juuls Vej 1, 2730 Herlev, Denmark; 2 Department of Radiology, Copenhagen University Hospital-Herlev and Gentofte, Herlev, Denmark; 3 Department of Clinical Medicine, Faculty of Health, and Medical Science, University of Copenhagen, Copenhagen, Denmark

**Keywords:** Acute disseminated encephalomyelitis, ADEM, COVID-19, SARS-CoV-2, Vaccine, Johnson & Johnson, Ad26.COV2.S

## Abstract

**Background:**

The severe acute respiratory syndrome coronavirus-2 (SARS-CoV-2) pandemic has been leading to dramatic health, social and economic problems around the world. It was necessary to introduce worldwide vaccination program against SARS-CoV-2 virus. Vaccination of billions of people around the world leads to many questions about risk of vaccines and possible side effects. It is well known that acute disseminated encephalomyelitis (ADEM) is a rare, but possible complication of vaccines. Previously, cases of ADEM following various COVID-19 vaccines, including the vaccines from AstraZenica, Pfizer, Sputnik V, SinoVac, Moderna, Sinopharm, have been described. In this case report, we present the first documented case of ADEM following the COVID-19 vaccine Ad26.COV2.S from Johnson & Johnson.

**Case presentation:**

We present the case of a 31-year-old female with gradually progression of right-sided weakness and numbness during a three-week period. Four weeks prior to symptom onset, the patient received the single-dose SARS-CoV-2 vaccine Ad26.COV2.S. Neuroimaging revealed five large juxtacortical T2 FLAIR hyperintense lesions with incomplete contrast enhancement on post-contrast T1 images located supratentorial: one in the right cerebral hemisphere and four in left cerebral hemisphere. The patient was followed up for four months. Symptom debut, clinical picture and MRI were typical for ADEM and the patient completely recovered after high dose intravenous methylprednisolone treatment.

**Conclusions:**

This is, to the best of our knowledge, the first case report of ADEM following the COVID-19 vaccine Ad26.COV2.S. This case illustrates, although ADEM is a rare complication following SARS-CoV-2 vaccines, the necessity of maintaining a vaccine safety monitoring system to identify patients at high risk from developing severe complications from the vaccines.

## Background

A variety of vaccines have been granted emergency use authorization to fight the severe acute respiratory syndrome coronavirus-2 (SARS-CoV-2) pandemic, also referred to as the COVID-19 pandemic. In the post-authorization phase, a broad range of neurological complications have been reported including neurovascular, neurometabolic and neuroinflammatory diseases (Garg and Paliwal 2022; Finsterer 2022). The reports of post-vaccination CNS inflammation included transverse myelitis, acute encephalitis, neuromyelitis optica and acute disseminated encephalomyelitis (ADEM).

ADEM is an autoimmune inflammatory disease of the CNS, characterized by a brief and widespread demyelination, predominantly involving the white matter, and often appears following a viral or bacterial infection.

We have been able to identify 17 case reports that have described ADEM, including one case report of ADEM-like presentation, following a COVID-19 vaccination: seven cases with mRNA vaccine (from Moderna and Pfizer), (Kania et al. 2021; Vogrig et al. 2021; Shimizu et al. 2021; Lee et al. 2022; Poli et al. 2022; Ahmad et al. 2022; Maramattom et al. 2022) seven cases with a viral vector vaccine (from AstraZenica and Sputnik V) (Rinaldi et al. 2022; Permezel et al. 2022; Mumoli et al. 2022; Nagaratnam et al. 2022; Al-Quliti et al. 2022; Lazaro et al. 2022; Garg et al. 2022) and three cases with vaccines based on inactivated virus (from SinoVac and Sinopharm) (Cao and Ren 2022; Ozgen Kenangil et al. 2021; Yazdanpanah et al. 2022). However, this case report is the first described case of ADEM following exposure to the Ad26.COV2.S vaccine from Johnson & Johnson.

## Case presentation

A 31-year-old Caucasian female, with no previous neurological history (Table 1—Patient characteristics), was admitted to the hospital due to slow progression of right-sided weakness and numbness during the past three weeks. At symptom onset, the patient suffered from headache, nausea, mild neck pain, and balance impairment, but no dizziness. Additionally, the patient and some of her family members had noticed mild behavioral changes and cognitive impairments including memory and concentration issues. These symptoms remitted spontaneously after 5–7 days. The patient reported no diarrhea, respiratory infections, fever or chills.

**Table 1:** Patient characteristics

Age	31 years
Sex	Female
BMI	31.6
Ethnic group	Caucasian
Medication	None
Medical history	No relevant history
Surgery history	Appendectomy—over 20 years ago
Family history	No relevant history

Four weeks prior to symptom onset, the patient received the single-dose SARS-CoV-2 vaccine from Johnson & Johnson, Ad26.COV2.S, a recombinant, replication-incompetent Ad26 vector, encoding a stabilized variant of the SARS-CoV-2 spike protein.

Neurological examination revealed moderate right-sided hemiparesis and hypoesthesia. No impaired consciousness was found. Blood tests including complete blood count; glucose; electrolytes; vitamins; thyroid-, kidney and liver function were all within normal reference ranges. Anti-aquaporin-4, anti-myelin oligodendrocyte glycoprotein, and antinuclear antibodies were negative. Screening for HIV and HCV was negative. HBV antigen was negative and HBV antibody was positive (HBs IgM + IgG), due to previous HBV vaccination.

Pharyngeal swab was negative for SARS-CoV-2 on reverse-transcriptase polymerase chain reaction assay. The cerebrospinal fluid (CSF) white blood cell count was 1 × 10^6^/L (reference range 0–5 × 10^6^/L); protein levels were 0.31 g/L (reference range 0.15–0.5 g/L); IgG index was 0.47 (reference range 0.38–0.67); and oligoclonal bands in CSF and blood were negative.

**Fig. 1:**
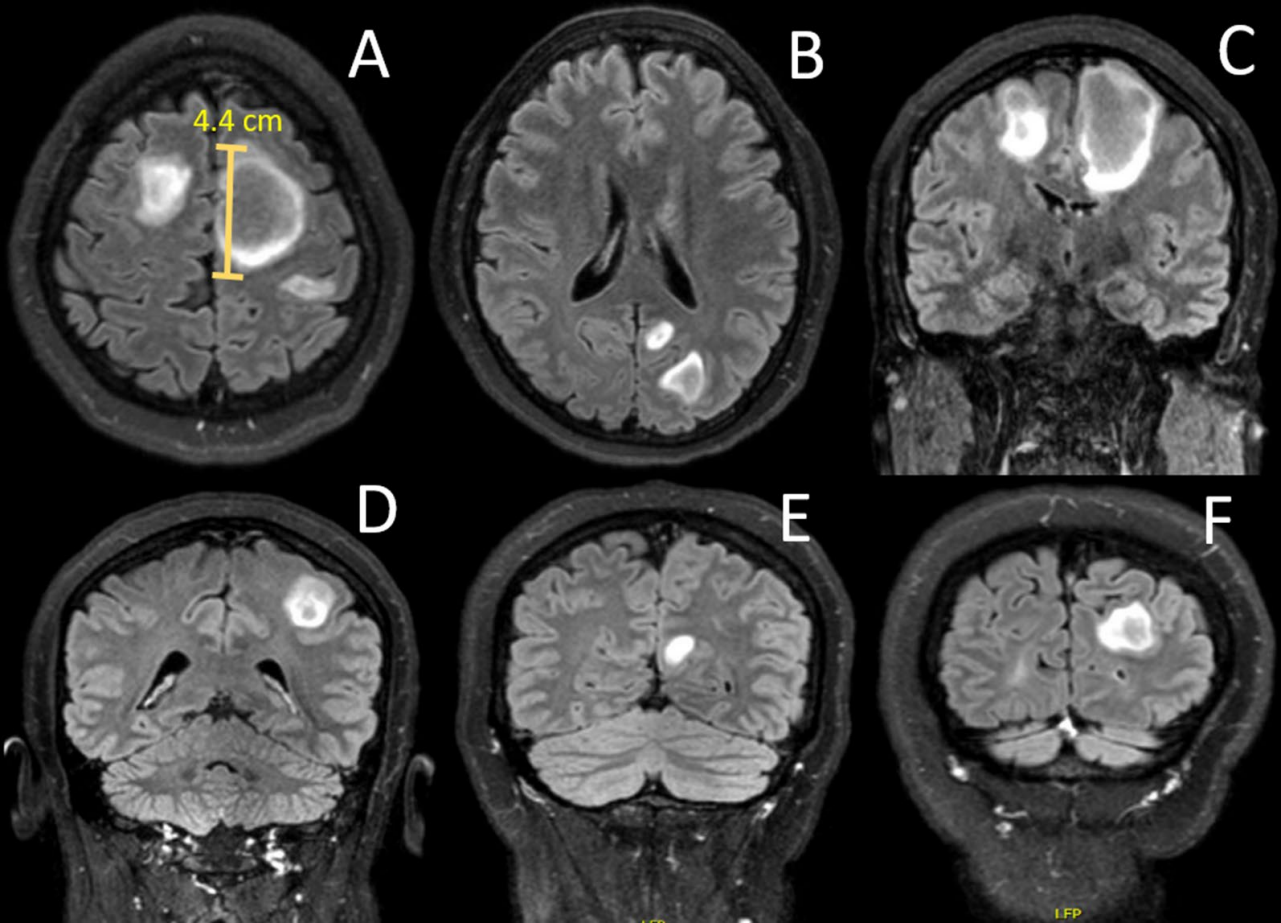
Axial T2 fluid-attenuated inversion recovery (T2 FLAIR) MRIs at admission showing five large juxtacortical lesions supratentorially. One lesion in the right cerebral hemisphere and four in the left cerebral hemisphere. The largest lesion was located in the left frontal lope and measured approximately 4.4 cm in diameter (**A**). **A** + **B** Axial sections. **C**–**F** Coronal sections

Brain and spinal cord magnetic resonance imaging (MRI) revealed five large juxtacortical T2 FLAIR hyperintense lesions with incomplete contrast enhancement on post-contrast T1 images located supratentorial: one in the right cerebral hemisphere and four in left cerebral hemisphere. The largest lesion was in the right frontal lope with a diameter of approximately 4.4 cm (Fig. 1). No infratentorial or spinal cord lesions were found. The clinical and radiological findings raised the suspicion of ADEM.

On the day at admission, the patient was treated with high-dose IV methylprednisolone, 1000 mg for three consecutive days followed by oral administration of 500 mg prednisolone for four days with significant clinical improvements. She was discharged from the hospital six days after admission where physical examination only revealed mild right-sided hemiparesis.

**Fig. 2:**
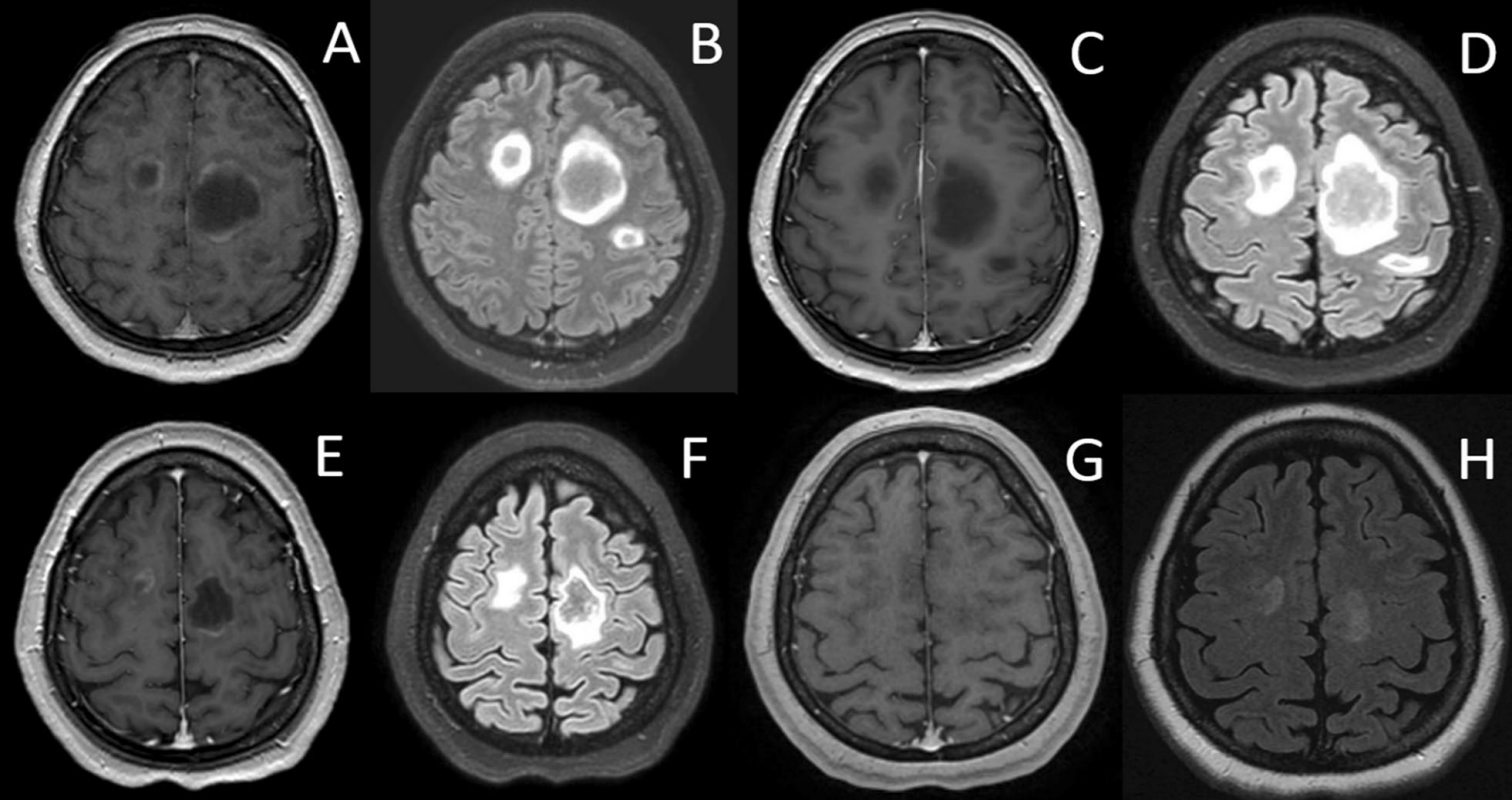
Post-contrast T1 weighted MRI (**A**, **C**, **E** and **G**) and axial T2 fluid-attenuated inversion recovery (T2 FLAIR) (**B**, **D**, **F** and **H**) during hospitalization and follow-up. First day at hospitalization (**A** + **B**), six days follow-up (**C** + **D**), five weeks follow-up (**E** + **F**) and four months follow-up (**G** + **H**)

A five-week follow-up MRI showed volume reduction in all the lesions, and no new lesions were found. Two lesions showed incomplete ring enhancement. The last MRI follow-up at four months showed regression in all five lesions with extensive lesion volume reduction and almost complete resolution (Fig. 2). Additionally, the patient presented with complete clinical recovery at the four-month follow-up.

## Conclusions

Many of the previous mentioned SARS-CoV-2 vaccines have been approved by federal agencies or national authorities, e.g., the U.S. Food and Drug Administration (FDA) or the European Medicines Agency (EMA) as it has been assessed that the overall benefits of the vaccines outweigh their risks. However, due to the extensive global use of SARS-Cov-2 vaccines. it is important to ensure a fast-acting safety monitor system to discover the rare complications to the vaccines, e.g., ADEM. This would improve the likelihood of identifying the right patients who are at high risk from developing severe complications from the vaccines.

The manifestation of the clinical and paraclinical findings in this case did meet the criteria for ADEM (Krupp et al. 2007). Our patient presented with multifocal neurological abnormalities including mild encephalopathy not explained by fever, postictal symptoms, or systemic illness. MRI of the brain showed diffuse, large (the largest at approximately 4.4 cm) lesions in the cerebral white matter and normal findings of the CSF including negative oligoclonal bands and normal IgG index. We saw a rapid clinical remission with high-dose corticosteroid treatment. It is well known that ADEM is associated with vaccines (i.e., measles, rubella or mumps). A large epidemiological study, which evaluated epidemiological features of post-vaccine ADEM, found that the time interval from vaccination to ADEM onset was 2–30 days in 61% of the cases (Pellegrino et al. 2013). In our case, the patient had symptom onset four weeks after vaccination, in line with the previously reported time-line from vaccine to onset of ADEM.

During the four-month follow-up, the clinical and paraclinical features followed a monophasic disease course; however, a prolonged observation is required to confirm this.

Overall, we have identified 17 previous case reports of patients with ADEM following SARS-CoV-2 vaccination, including vaccines based on mRNA, viral vector and inactivated virus (Kania et al. 2021; Vogrig et al. 2021; Shimizu et al. 2021; Lee et al. 2022; Poli et al. 2022; Ahmad et al. 2022; Maramattom et al. 2022; Rinaldi et al. 2022; Permezel et al. 2022; Mumoli et al. 2022; Nagaratnam et al. 2022; Al-Quliti et al. 2022; Lazaro et al. 2022; Garg et al. 2022; Cao and Ren 2022; Ozgen Kenangil et al. 2021; Yazdanpanah et al. 2022); however, this case report is the first described case of ADEM following exposure to the Ad26.COV2.S vaccine.

In previous cases, the patients age ranged from 19 to 88 years but were predominantly in the ages between 30 and 50, and the majority was females, which is in line with our case. In contrast, the previous cases primarily had time from vaccination to symptoms of 10–14 days, which is half the time as the patient in our case. Additionally, most patients from the previous cases, similar to the patient in our case, had no previous significant medical history, and none of the patients were treated with immune-modifying drugs. This illustrates, although ADEM is a rare complication following SARS-CoV-2 vaccines, the necessity of maintaining a vaccine safety monitoring system to identify patients at high risk from developing severe complications from the vaccines.

## Data Availability

The data that support the findings of this case report are available from the corresponding author, [SG], upon reasonable request.

## Abbreviations

ADEM: Acute disseminated encephalomyelitis
CSF: Cerebrospinal fluid
HBV: Hepatitis B-virus
HCV: Hepatitis C-virus
HIV: Human immunodeficiency virus
MRI: Magnetic resonance imaging
SARS-CoV-2: The severe acute respiratory syndrome coronavirus-2
T2 FLAIR: T2 fluid-attenuated inversion recovery
